# Improvement on the genetic engineering of an invasive agricultural pest insect, the cherry vinegar fly, *Drosophila suzukii*

**DOI:** 10.1186/s12863-020-00940-5

**Published:** 2020-12-18

**Authors:** Hassan M. M. Ahmed, Fabienne Heese, Ernst A. Wimmer

**Affiliations:** 1 Department of Developmental Biology, Johann-Friedrich-Blumenbach-Institute of Zoology and Anthropology, Göttingen Center for Molecular Biosciences, Georg-August-University Göttingen, 37077 Göttingen, Germany; 2 Department of Crop Protection, Faculty of Agriculture-University of Khartoum, P.O. Box 32, 13314 Khartoum North, Khartoum Sudan

**Keywords:** Binary expression system, Enhancer/promoter, Insect transgenesis, Molecular entomology, Pest management, Spotted wing *Drosophila*, Sterile insect technique

## Abstract

**Background:**

The invasive fly *Drosophila suzukii* has become an established fruit pest in Europe, the USA, and South America with no effective and safe pest management. Genetic engineering enables the development of transgene-based novel genetic control strategies against insect pests and disease vectors. This, however, requires the establishment of reliable germline transformation techniques. Previous studies have shown that *D. suzukii* is amenable to transgenesis using the transposon-based vectors *piggyBac* and *Minos*, site-specific recombination (*lox*/Cre), and CRISPR/Cas9 genome editing.

**Results:**

We experienced differences in the usability of *piggyBac*-based germline transformation in different strains of *D. suzukii*: we obtained no transgenic lines in a US strain, a single rare transgenic line in an Italian strain, but observed a reliable transformation rate of 2.5 to 11% in a strain from the French Alps. This difference in efficiency was confirmed by comparative examination of these three strains. In addition, we used an *attP* landing site line to successfully established φC31-integrase-mediated plasmid integration at a rate of 10% and generated landing site lines with two *attP* sequences to effectively perform φC31-Recombinase Mediated Cassette Exchange (φC31-RMCE) with 11% efficiency. Moreover, we isolated and used the endogenous regulatory regions of *Ds nanos* to express φC31 integrase maternally to generate self-docking lines for φC31-RMCE. Besides, we isolated the promoter/enhancer of *Ds serendipity α* to drive the heterologous *tetracycline-controlled transactivator (tTA*) during early embryonic development and generated a testes-specific tTA driver line using the endogenous *beta-2-tubulin* (*β2t*) promoter/enhancer.

**Conclusion:**

Our results provide evidence that the *D. suzukii* strain AM derived from the French Alps is more suitable for *piggyBac* germline transformation than other strains. We demonstrated the feasibility of using φC31-RMCE in the cherry vinegar fly and generated a set of lines that can be used for highly efficient integration of larger constructs. The φC31-based integration will facilitate modification and stabilization of previously generated transgenic lines that carry at least one *attP* site in the transgene construction. An early embryo-specific and a spermatogenesis-specific driver line were generated for future use of the binary expression system *tet-off* to engineer tissue- and stage-specific effector gene expression for genetic pest control strategies.

**Supplementary Information:**

The online version contains supplementary material available at 10.1186/s12863-020-00940-5.

## Background

The invasive pest *Drosophila suzukii* commonly referred to as the cherry vinegar fly or the spotted wing *Drosophila* (SWD) originated from East Asia [1, 2]. It was described for the first time in Japan in 1916. In 2008, the fly has concomitantly been reported in Europe (Spain and Italy) and the USA (California), where the SWD presents a major threat to the soft and stone fruit industry [1–3]. The fly is armed with a prominent serrated ovipositor that enables it to lay eggs inside ripening intact fruits. The larvae eat and develop inside the fruits and lead to a crop loss of up to 100% [4]. Several insecticides have been used to control the fly with limited degrees of success [5, 6]. A genetic control method, the Sterile Insect Technique (SIT), might provide the most promising pest management strategy. SIT was proposed more than 75 years ago as biological control method to fight agricultural pests and diseases vectors. It is a species-specific birth control strategy, which makes it safe for pollinators and natural enemies and is thus environmentally friendly [7]. The SIT consists of mass rearing of the target pest in large numbers, sexing, sterilization of the males and successive inundative release in the target area. Genetic engineering offers different approaches for improvement of SIT [8–13]. For example, a transgene-based conditional embryonic lethality system was developed as a way to induce reproductive sterility, which can replace the need for ionizing radiation and ensure production of competent males [9, 10]. A transgenic female-specific embryonic lethality system developed for several dipterans, notably tephritid fruit flies, serves a method to eliminate females during early embryonic development and facilitates the production of only males for SIT releases [11–14].

The ability to genetically manipulate biological systems from mammalian and insect cell lines to insects and mouse has been revolutionized by the discovery and utilization of the most versatile transposon, *piggyBac* [15–17]*.* It belongs to the class II DNA transposons, which work by a cut and paste mechanisms [18]. *piggyBac*-based vectors were generated to insert cargo sequences at a *TTAA* recognition sequence in the genome of the target species. *piggyBac*-based germline transformation has been successfully established for many model and no-model organisms including *Drosophila melanogaster* [19–21], *Ceratitis captitata* [22, 23], *Anastrepha suspensa* [24], *Drosophila suzukii* [25], *Anopheles gambiae* [26], *Aedes aegypti* [27], *Musca domestica* [28], among others. The increase in the efficiency of germline transformation due to the use of a hyperactive version of the *piggyBac* transposase was demonstrated in several insects [23]. An inherent characteristic of transposon vectors using *piggyBac* is the random integration in the genome which makes them a useful tool for mutagenesis screens, enhancer traps, and exon traps [19, 29–31]. Also, in cases, when no clear target sequence can be identified, the random integration might result in a set of insertions, from which to choose the most suitable ones. However, this randomness is considered a drawback, when different transgenes were to be compared in the same genomic context [32, 33].

Site-specific recombinases (SSR) offer a more precise approach for genetic engineering of biological systems [34, 35]. In the presence of the respective recombinase, recombination takes place between two identical sequences in case of Flp/*FRT* and Cre/*lox* [36, 37] or non-identical sequences in case of φC31 *attP*/*attB* [38]. The use of SSR necessitates the generation of landing site lines by integrating at least a single landing site (*FRT*, *lox* or *attP*) into the genome of the target species. This is routinely done by including the sequence within a transposon vector and integrate it randomly in the genome. Once generated, these landing sites can be used repeatedly to integrate different transgenes. In case a single landing site is integrated, the transgene of interest has to be delivered in a plasmid vector that has the respective recombinase recognition sequence which leads to integration of the whole plasmid including the antibiotic resistance gene. To avoid this, two landing sites can be placed close to each other into the genome ideally separated by a marker. The transgene to be inserted has to be flanked by two recombinase recognition sequences, which facilitate double recombination events leading to a recombinase mediated cassette exchange (RMCE). The φC31-based integration and RMCE have been established in many insects for either modification and or stabilization of previously generated transgenes [39] or for site-specific germline transformation, which allows examination of different transgenes in the same genomic context [40]. Furthermore, the use of the φC31 system allows for large transgenes to be integrated. In fact, BAC constructs of up to 133 kb were integrated using this system [41]. Moreover, in *Drosophila* and mosquitoes the φC31 system has been used to generate self-docking strains that expresses the integrase from the enhancer/promoter of the maternal effect gene *nanos*. This has remarkably improved the efficiency of site-directed germline transformation [42, 43].

To generate transgene-based reproductive sterility or sexing strains, food supplement-controlled binary expression systems have widely been used for conditional and tissue- or stage-specific gene expression [8–14]. The UAS/Gal4 system has intensively been used in *D. melanogaster* to drive tissue-specific expression of dsRNA to knockdown genes and study their function [44, 45]. The tet system has initially been developed to be used in human cell culture and has since been engineered into *tet-off* and *tet-on* systems [46–48]. In insect biotechnology, the *tet-off* system was used e.g. to control the expression of effector molecules such as the proapoptotic gene *head involution defective* (*hid*), which leads to apoptotic cell death [8]. To drive the heterologous transactivator of such a binary expression system to cause effective reproductive sterility [9, 10] or female-specific killing [11, 13, 14, 49] based on early embryonic lethality, the promoter/enhancers of cellularization-specific genes need to be identified and isolated. Moreover, to direct sperm-specific expression for transgenic marking [50–52] or the development of multifactorial reproductive sterility [53], the use of promoters/enhancers active during spermatogenesis are of interest.

Here we show that *D. suzukii* strains originated from different locations can be transformed using *piggyBac* germline transformation with varying efficiency. In addition, we demonstrate the successful use of φC31-based site-specific germline transformation both by integration in one *attP* site or by RMCE. Moreover, we provide a set of *D. suzukii* self-docking lines expressing φC31 integrase maternally during oogenesis. Furthermore, we provide an early embryo-specific and a spermatogenesis-specific driver line for using the *tet-off* binary expression system to drive tissue-specific expression of effector genes.

## Results

### Comparison of *piggyBac* germline transformation in different *D. suzukii* strains

**Fig. 1:**
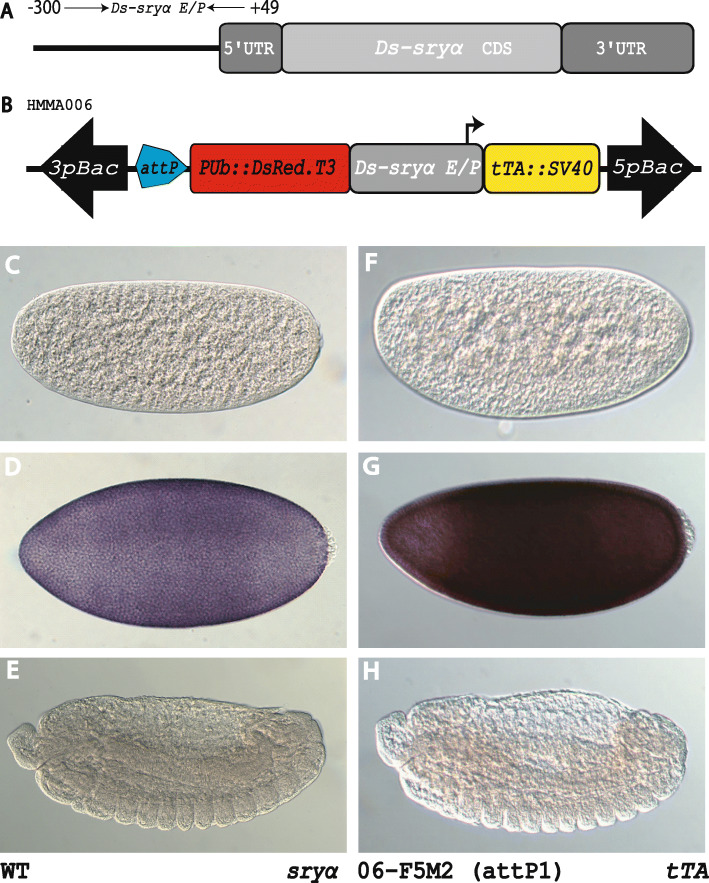
*D. suzukii serendipity α* and the use of its promoter/enhancer for directed expression. **a** Schematic representation of the *D. suzukii* gene *serendipity α*. **b** *piggyBac*-based transgenic construct HMMA006 [52] to drive *tTA* during early embryonic development. **c-e** Whole mount in situ hybridisation to detect *Ds_sry α* expression in wildtype *D. suzukii* embryos. **f-h** Whole mount in situ hybridisation to detect *tTA* expression in transgenic *D. suzukii* embryos of line 06_F5M2 (attP#1) carrying construct HMMA006. **c,f** Syncytial blastoderm embryos before start of cellularization. **d,g** Syncytial blastoderm embryos during cellularization show expression of *sry α* or *tTA*, respectively. **e,h** Germ band retracting embryos

**Fig. 2:**
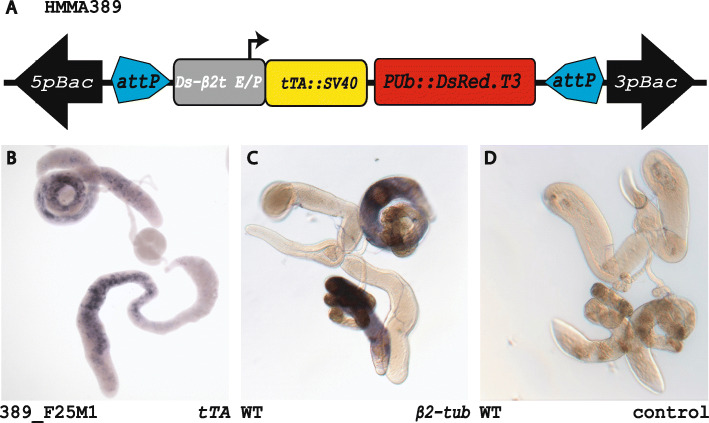
Spermatogenesis-specific driver for binary *tet-off* expression system. **a** *piggyBac*-based transgenic construct HMMA389 to generate a testes-specific driver line carrying the *β2t* promoter [52] fused to *tTA*. **b-d** Whole mount in situ hybridisation to detect gene expression in *D. suzukii* male reproductive organs. **b** Testes-specific *tTA* expression driven by the *Ds_β2t* promoter in line 389_F25M1. **c** *Ds_β2t* expression in wildtype testes detected by an antisense probe. **d** Negative control using a *Ds_β2t* sense probe on wildtype testes

**Fig. 3:**
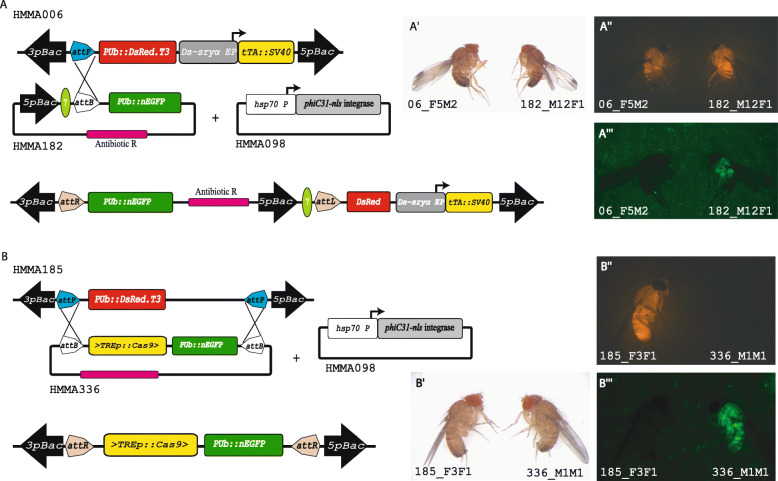
φC31-mediated site-specific integration and RMCE. **(A)** Scheme for site-specific germline transformation. *D. suzukii* line 06_F5M2 [52] carries construct HMMA006 that contains an *attP* recombination target sequence, which - in the presence of a helper plasmid providing φC31 integrase (HMMA098) - is targeted by construct HMMA182 carrying the corresponding *attB* recombination site to integrate the complete plasmid. The integration leads to a modification of the transgenic insert, which can be used for additional integration of transgenes (light green “?”) as well as transgene stabilization by removing part of the transgenic composition by *piggyBac* excision [39]. (**A′-A‴**) Integration can be detected by the addition of the EGFP marker. **(B)** RMCE to generate diverse transgenes at the same genomic position. *D. suzukii* line carrying construct HMMA185 is targeted by construct HMMA336 in the presence of a helper plasmid (HMMA098) providing φC31 integrase to exchange marker genes and integrate a specific cargo gene (*TRE-Cas9*). (**B′-B‴**) RMCE can be detected by the replacement of the DsRed marker with the EGFP marker. Images of a male fly of each indicated line are taken with cold light (**A′,B′**), RFP filter (**A″,B″**), or EYFP filter (**A‴,B‴**)

**Fig. 4:**
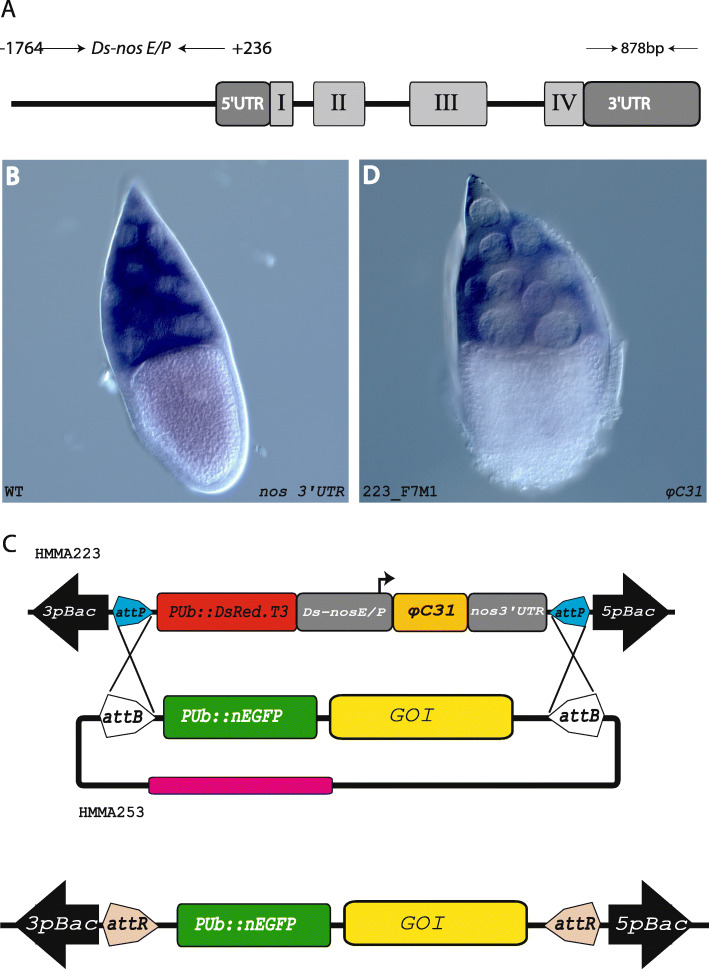
*D. suzukii nanos* and the use of its promoter/enhancer for directed expression. **a** Schematic representation of the *D. suzukii* gene *nanos*. **b** Whole mount in situ hybridisation to detect *nanos* expression in wildtype *D. suzukii* ovaries. **c** *piggyBac*-based transgenic construct HMMA223 to generate φC31 integrase RMCE self-docking lines. RMCE in a self-docking line, which provides both the recombination target sequences *attP* as well as the φC31 integrase driven by the *nanos* promoter/enhancer providing maternal expression, will result in marker exchange as well as cargo gene (*GOI*) integration and removal of the integrase source. **d** Whole mount in situ hybridisation to detect *φC31 integrase* expression in transgenic *D. suzukii* ovaries carrying construct HMMA223. Expression of *nanos* or *φC31 integrase*, respectively, is detected in the nurse cells of the ovaries

Transposon-based vectors have been intensively used for genetic manipulation from cell culture to mouse. The vector *piggyBac* has gained particular attention due to its versatility and usability in different systems. When we started to use *piggyBac* for germline transformation of an Italian strain of *D. suzukii*, we had only poor success and retrieved a rare transgenic line (06_F5M2) carrying construct HMMA006 [52], which mediates early embryonic expression of *tTA* (Fig. 1), with a transformation rate of 1.6% (300 embryos injected, 200 survived, 60 fertile, 1 transgenic line). However, several previous attempts with the same construct and additional attempts with five other constructs were unsuccessful. Changing to a US strain did not improve our approach, since trying the same five different constructs in this strain did not yield any transgenic lines. Only once we changed to the strain Alpes Maritimes (AM) isolated from the French Alps [54], we started to get reliable *piggyBac* germline transformation to work. In this strain, we regularly obtained transgenic lines for three different constructs with transformation rates between 2.5 and 11% (Additional file 1): The testes-specific driver construct HMMA389, which is designed to be also useable for φC31-mediated RMCE and mediates spermatogenesis-specific expression of *tTA* (Fig. 2); the DsRed-marked construct HMMA185 containing two *attP* sites for φC31-mediated RMCE (Fig. 3); as well as the construct HMMA223 to generate self-docking lines for φC31-mediated RMCE (Fig. 4). Additional file 2 provides a list of the obtained lines.

To examine the suitability of the three different strains for *piggyBac* germline transformation in a truly comparative manner, we injected construct HMMA223 to generate more self-docking lines for φC31-mediated RMCE (Fig. 2c) into similar amounts of embryos on the same day and with the same injection needle to minimize variations in the injection procedure. Table 1 shows that no transgenic lines were obtained with the US or Italian strains, but were successfully obtained with the French AM strain with a transformation rate of 4.2%. This demonstrates the higher usability of the AM strain for *piggyBac* germline transformation.

**Table 1:** Comparative *piggyBac* transformation efficiency in different *D. suzukii* strains

Origin of *D. suzukii* strain	No. of injected embryos	Hatched larvae	Fertile crosses	No. of transgenics	Transformation rate in %
Italy	400	190	35	0	–
France (AM)	450	210	47	2	4.2
USA	430	240	50	0	–

### Isolation of an enhancer/promoter region active during early embryonic development

To direct gene expression specifically at early embryonic development, we identified the *serendipity α* (*sry α*) gene by homology search in the *D*. *suzukii* genome database (www.spottedwingflybase.org) using the *Dm_sry α* sequence as query. The open reading frame of the *Ds_sry α* gene from the translation start codon to the stop codon is 1593 bp without introns. The gene has a 5’UTR of 49 bp, which demarcates the transcription start site (Fig. 1a). The *Ds_sry α* coding sequence encodes a putative protein of 530 amino acids, which shares 86% identity to Dm_Sry α protein.

To validate the cellularization-specific expression of the isolated *Ds_sry α* gene, we performed whole mount in situ hybridization on different stage wild type embryos using a DIG-labelled antisense probe against the whole *Ds_sry α* ORF plus the 5′ UTR. These in situ hybridizations detected expression only during blastoderm cellularization with no expression at earlier or later embryonic stages (Fig. 1c-e).

To identify the necessary upstream and downstream regulatory elements driving cellularization-specific gene expression, we compared the *Ds_sry α* sequence with the characterized counterpart in *D. melanogaster* [9]. To examine, whether the 300 bp upstream regulatory element plus the 49 bp 5’UTR drive cellularization-specific gene expression, we fused this 349 bp enhancer/promoter fragment of the *Ds_sry α* gene to *tTA* (Fig. 1b) and generated *D. suzukii* line 06_F5M2 [52] by *piggyBac*-based germline transformation. Embryos from this line were then tested by whole mount in situ hybridization for expression of *tTA*, which revealed the respective cellularization-specific expression pattern of *Ds_sry α* (Fig. 1f-h) indicating that the isolated promoter/enhancer element is suitable for stage-specific gene expression during early embryonic development.

### Spermatogenesis-specific driver for binary *tet-off* expression system

Since direct expression of effector molecules potentially causing harm obstructs the generation of transgenic lines, we aim to establish the *tet-off* binary system in *D. suzukii* to develop transgenic improvements for SIT approaches. To examine this binary expression system, we used the *Ds_β2t* enhancer/ promoter [52] to generate construct HMMA389 (Fig. 2a). By *piggyBac*-based germline transformation, we obtained the spermatogenesis-specific driver line 389_F25M1 that expresses *tTA* in the testes. The spermatogenesis-specific expression was confirmed by in situ hybridization and compared to the endogenous expression of *Ds_β2t* (Fig. 2b-d).

### φC31-mediated site-specific germline transformation

Modification and/or stabilization of transgenes generated by transposon-based vectors by site-specific recombination have been demonstrated in *D. melanogaster* and *Ceratitis capitata* [39, 55]. To establish φC31-based site-specific germline transformation by integration of a transgene construct into a single *attP* site, we injected donor plasmid HMMA182 carrying an *EGFP* transformation marker and the bacterial attachment sequence *attB* along with helper plasmid HMMA098 expressing φC31 integrase under the promoter of the *Ds-hsp70* gene into pre-blastoderm embryos of the DsRed-marked transgenic embryonic driver line 06_F5M2 (attP#1). This line was generated with construct HMMA006 [52], which harbours in addition to the early embryonic tTA-driver also an *attP* site (Figs. 1b, 3A). Out of 250 injected embryos 110 hatched and 40 fertile G_0_ crosses gave rise to four independent integrations (Additional file 2), which were identified by showing both red and green fluorescent markers (Fig. 3A′-A‴), resulting in a site-specific transformation efficiency of 10%.

### φC31-mediated recombination mediated cassette exchange

To examine a docking line with two *attP* sites in opposite orientation of a DsRed-based transformation marker for establishment of RMCE in *D. suzukii*, we used the docking line 185_F3F1 (RMCE#1), which resulted from *piggyBac*-mediated integration of vector HMMA185 (Fig. 3B) into the AM strain (Additional files 1 and 2). In this line, we confirmed the presence of the two *attP* sites by sequencing. To see whether the φC31-based RMCE works in *D. suzukii*, we co-injected into this line plasmid HMMA336 having two *attB* recombination sites in opposite orientation flanking an EGFP-based transformation marker and the transgene of interest (an effector to drive *Cas9* expression under the control of the binary expression system *tet-off*) along with the helper plasmid HMMA098 (Fig. 3B). We obtained 71 G_0_ fertile crosses, of which eight gave rise to F_1_ progeny that showed EGFP and absence of DsRed fluorescence (Fig. 3B′-B‴) indicating an RMCE rate of 11,3%. RMCE line 336_F3F2 was then used to verify the faithful double recombination event by PCR and sequencing of the resulting hybrid *attL* and *attR* sites (Fig. 3B).

### Isolation of an enhancer/promoter region active during oogenesis and in the germline to generate self-docking lines for φC31-mediated RMCE

To improve φC31-mediated RMCE further, we wanted to establish self-docking lines (Fig. 4) that express φC31 integrase maternally in addition to carrying two *attP* recombination sites. In this respect, we identified the *Ds_nanos* gene by homology search in the *D*. *suzukii* genome database (www.spottedwingflybase.org) using the *Dm_nanos* sequence as query. The open reading frame of the *Ds_nanos* gene from the translation start codon to the stop codon is 2433 bp, which is interrupted by three introns. The gene has a 5’UTR of 236 bp, which demarcates the transcription start site and a 3′ UTR of 878 bp (Fig. 4a). To validate the oogenesis- and germline-specific gene expression of the isolated *Ds_nanos* gene, we performed whole mount in situ hybridization on ovaries using DIG-labelled antisense probes against the *Ds_nanos* 3’UTR and 103 bp of exon IV. These in situ hybridizations detected expression in ovarian nurse cells (Fig. 4b).

To identify the necessary upstream and downstream regulatory elements driving oogenesis-specific gene expression, we compared the *Ds_nanos* sequence with the characterized counterpart in *D. melanogaster*. To examine, whether the 2 Kb enhancer/promoter region including the 5’UTR drives oogenesis-specific gene expression, we fused this 2 Kb enhancer/promoter fragment of the *Ds_nanos* gene to the coding region of *φC31 integrase* (Fig. 4c) and generated *D. suzukii* lines 223_F7M1 and 223_M3M2 by *piggyBac*-based germline transformation of the AM strain (Additional files 1 and 2). In addition, two more self-docking lines were generated in the comparative approach to evaluate the different *D. suzukii* strains (Table 1). Ovaries from line 223_M3M2 were then tested by whole mount in situ hybridization for expression of *φC31 integrase*, which revealed the respective nurse cell-specific expression in the ovaries (Fig. 4d) resembling *Ds_nanos* expression, which indicates that the isolated promoter/enhancer element is suitable for maternal gene expression.

## Discussion

The discovery that exogenous DNA can be stably introduced into the germline of living organisms which can then be stably inherited by the offspring has tremendously contributed to the advancement of biological and biomedical research and in particular functional genetic studies [15, 19, 30, 31]. The road for insect genetic engineering has been well paved by geneticists working with the model organism *D. melanogaster*. Genetic screens in *D. melanogaster* using *P-element* based transformation vectors to perform insertional mutagenesis, enhancer- and gene-traps, as well as ectopic or overexpression studies provided an enormous contribution to our understanding of gene function [56–58]. Unfortunately, the *P-element* is not functional in other organisms due to the requirement of host-specific factors [59]. Transformation vectors based on the lepidopteran transposable element *piggyBac* have been used to engineer many insects [20, 22, 25, 26] and encouraged the establishment of new insect model systems such as *Tribolium castaneum* [60, 61].

The invasive fruit pest, *D. suzukii*, had been successfully transformed using *piggyBac*-based vectors [25, 54, 62]. We have used three different lab strains of *D. suzukii* from Italy, USA, and France. After many attempts to generate transgenic *D suzukii* using *piggyBac* germline transformation with different constructs by microinjection into the Italian strain, we obtained only one transgenic line, 06_F5M2, with a low transformation efficiency of 1.6% and failed to obtain any transgenic flies from the US strain. When we obtained the French strain AM (which was kindly provided to us by N. Gompel, Munich), we managed to get reliably transgenics with varying efficiency. Based on these observations and the comparative examination of these three strains (Table 1), we recommend the AM strain for *piggyBac* germline transformation.

Due to the random integration of transposon-based transformation vectors and the limited size of cargo they can carry, we decided to extend the toolkit for *D. suzukii* transformation by firmly establishing a site-specific transformation technology. Recombinase-based site-specific germline transformation such as (Cre/*lox*, flp/*FRT* and φC31 *attP*/*attB*) had been established in many model and non-model insects and shown to overcome the shortcomings of transposon-based germline transformation [40]. The Cre/*lox* Recombinase Mediated Cassette Exchange has recently been established for the cherry vinegar fly *D. suzukii* [63]. In this study, we demonstrate the feasibility of using the *φC31* integrase system to integrate a construct in a single *attP* landing site. This approach had previously been established for *D. melanogaster* and the Mediterranean fruit fly *Ceratitis capitata*, where it was used to modify transgenic lines generated by random transposon-based vectors and to stabilize the transgene by subsequent deletion of one of the inverted repeats required for transposition [39, 55]. In addition, we have generated a docking line with two *attP* sites in opposite orientations and show that φC31-mediated RMCE works in *D suzukii*. The use of an endogenous source of φC31 integrase by expression from a germline specific enhancer/promoter had been shown to increase the efficiency of φC31-mediated integration and RMCE [42, 43]. In this regard, we set to generate self-docking lines that express *φC31 integrase* maternally. We isolated the endogenous *Ds-nanos* gene (Fig. 4) in order to use the upstream enhancer/promoter and the downstream 3’UTR for directing the expression of *φC31 integrase* to the nurse cells for maternal contribution to the early embryo. By random *piggyBac* germline transformation, we generated four transgenic lines with a *DsRed* body marker and the *φC31 integrase* cassette flanked by *attP* sites.

To be able to conditionally drive expression of effector genes in a tissue- or stage-specific manner, a suppressible or inducible binary expression system is required. This has been successfully exploited to develop biotechnological pest control strategies such as early embryonic lethality or female-specific embryonic lethality [9–14]. To develop such transgenic pest control strategies for the invasive pest *D. suzukii*, we identified a gene that is active during early embryonic stages (*Ds_sry α*) and a gene that is spermatogenesis-specific (*Ds_β2t*) [52]. 350 bp upstream regulatory sequence of the *Ds_sry α* gene were identified to be sufficient to drive the expression of *tTA* specifically during cellularization similar to the endogenous gene. This driver line will be usable to generate reproductive sterility or sexing lines by driving expression of pro-apoptotic genes as previously described for several tephritid fruit pests [10–12, 14]. Such systems will be very important to establish SIT programs for the control of this invasive pest species. In addition, we were able to generate a spermatogenesis specific driver line using the promoter of the *Ds_β2t* gene described previously [52].

## Conclusion

By comparing different *D. suzukii* strains for their usability for *piggyBac*-based germline transformation, we could clearly identify the AM strain derived from the French Alps as the most suitable one. In addition, we demonstrated that φC31-based site-specific integration and RMCE can be used routinely in the cherry vinegar fly, *D. suzukii*, and generated four self-docking lines for RMCE. The φC31-based integration will facilitate efficient integration of larger transgenic constructs and allow for the modification and stabilization of previously generated transgenic lines that carry at least one *attP* site in the transgene construction.

## Methods

### *Drosophila suzukii* strains

All fly experiments were performed in our well-equipped safety level one (S1) laboratory, which is certified for generating and using genetically modified insects. Wild type *D. suzukii* from Italy, USA (both kindly provided by Prof. Marc F. Schetelig), and French Alps (Prof. Dr. Nicolas Gompel) as well as the generated transgenic flies were reared on standard *Drosophila* food and kept at 25 °C throughout this study.

### Nucleic acid isolation

Genomic DNA isolation was done from a mix of adult males and females using NucleoSpin® DNA Insect (Macherey-Nagel) according to the manufacturer instructions. Total RNA was isolated from 0 to 24 h embryos enriched for 0–4 h stages using ZR Tissue & Insect RNA MicroPrep (Zymo Research Europe, 79110 Freiburg) according to manufacturer instructions.

All PCR amplifications during the course of this study were performed using Phusion DNA polymerase and Phusion-HF buffer (New England Biolabs GmbH, D-65926 Frankfurt am Main). A list of the used primers is provided in Additional file 3. Plasmid min-preps and PCR products were purified using NucleoSpin® Plasmid and NucleoSpin® Gel and PCR Clean-up kits (Macherey-Nagel GmbH & Co., 52355 Dueren, Germany), respectively. NucleoSpin® Plasmid Transfection-grade (Macherey-Nagel) or QIAGEN Plasmid Plus Midi Kit (QIAGEN GmbH, 40724 Hilden, Germany) were used to prepare plasmids for germline transformation.

### Amplification of cDNA ends

To isolate the 5’UTR and the 3’UTR of the early embryonic gene *Ds*_*sryα* and the maternal effect gene *Ds_nanos*, total RNA from 0 to 24 h old (enriched for 0-4 h) *D. suzukii* embryos was isolated and 1.3 μg were used to generate 5′ RACE-ready cDNA or 3’RACE-ready cDNA using SMARTer™ RACE cDNA amplification kit (Takara Bio Europe SAS, 78100 Saint-Germain-en-Laye, France) according to manufacturer instructions.

The 5’UTR of *Ds_sryα* and *Ds_nanos* were recovered by RACE PCR using gene specific primers HM#34 and HM#76, respectively, along with the universal primer (UPM) provided with the kit using Advantage2 DNA polymerase (Takara) with the following program: 94 °C 2 min, (94 °C 30 s, 72 °C 3 min) 5X, (94 °C 30 s, 70 °C 30 s, 72 °C 3 min) 5X, (94 °C 30 s, 68 °C 30 s, 72 °C 3 min) 30X. A single prominent band for each gene was recovered, purified, cloned into pCRII (Thermo Fisher Scientific) to generate pCRII_sryα_5’UTR (HMMA001) and pCRII_nos_5UTR (HMMA012), and sequenced using standard M13 primers.

To recover the 3’UTR of *Ds_sryα* and *Ds_nanos,* the gene specific primers HM#42 and HM#77, respectively, along with UPM provided with the kit using Advantage2 DNA polymerase (Takara) were used with the following program: 94 °C 2 min, (94 °C 30 s, 72 °C 3 min) 5X, (94 °C 30 s, 70 °C 30 s, 72 °C 3 min) 5X, (94 °C 30 s, 68 °C 30 s, 72 °C 3 min) 30X. A single prominent band for each gene was recovered, purified, cloned into pCRII (Thermo Fisher Scientific) to generate pCRII_sryα_3UTR (HMMA002) and pCRII_nos_3UTR (HMMA013), and sequenced using standard M13 primer.

### Plasmids construction

The plasmid HMMA020 was generated by PCR amplification of the coding sequence of *D. suzukii sryα* gene plus the 5’UTR using primer pair HM#16/HM#17 and advantage 2 DNA polymerase (Invitrogene) with program 98 °C 3′ followed by [98 °C 30″, 55 °C 30″,72 °C 2′]35X and cloned into the pCRII vector (Invitrogene).

To generate plasmid HMMA021 for in vitro synthesis of RNA probes, the *tTA* coding sequence was excised from mfs#1215 [10] using *Eco*RV/*Bam*HI and cloning into pCRII vector digested by the same enzymes.

To generate plasmid HMMA339 for in vitro synthesis of RNA probe against *φC31* integrase mRNA, 800 bp of the coding sequence was digested out from plasmid HMMA98 using *Sma*I/*Not*I and cloned into pCRII plasmid digested by *Eco*RV/*Not*I.

The plasmid FCMH01 was generated by PCR amplification of 800 bp of *Cas9* coding sequence using primers pair HM#560/HM#561 with program 98 °C 3′ followed by [98 °C 30″, 64 °C 30″, 72 °C 30″] 5X [98 °C 30″, 72 °C 1′] 35X, digested by and cloned into *Xho*I/*Bam*HI sites of pCRII vector.

To generate *piggyBac* transformation vector HMMA185 and HMMA186, first plasmid HMMA006 [52] was digested by *Asc*I to remove *sry*α*-tTA*, and the backbone was ligated to give rise to HMMA007. *attP220* was PCR amplified from HMMA007 using primer pair HM#368/HM369 and program 98 °C 3’followed by [98 °C 30″, 58 °C 30″, 72 °C 20″] 5X [98 °C 30″,72 °C 1′] 35X and cloned into *Eco*RV cut site of HMMA007 to give rise to HMMA185. To generate HMMA186 the *Eco*RI/*Hpa*I fragment *PUb::nlsEGFP* from mfs#1213 [51] was cloned into the *Eco*RI/*Hpa*I sites of HMMA185.

For the generation of *piggyBac* transformation vectors HMMA330 and HMMA331, first Gibson assembly was performed to clone *EGFPSV40* and the *3XP3* promoter into the *piggyBac* backbone of HMMA007 digested by *Eco*RI to give rise to HMMA227, in which the *EGFP* gene was then replaced by *DsRed.T3* from HMMA007 by *Age*I/*Not*I to give rise to HMMA228. Then the *attP220* was PCR amplified from HMMA007 using primer pair HM#131/HM#117 with PCR program 98 °C 3’followed by [98 °C 30″, 60 °C 30″, 72 °C 20″] 35X and cloned into *Eco*RI site of HMMA227 and HMMA228 giving rise to HMMA304 and HMMA305, respectively. Finally, the *Asc*I/*Age*I fragments from mfs#1213 and mfs#1214 [51] containing the *PUb* promoter were cloned into *Asc*I/*Age*I sites of HMMA304 and HMMA305 to give rise to HMMA330 and HMMA331, respectively.

To generate the spermatogenesis specific driver construct HMMA389, 1 kb upstream region of *D. suzukii Ds*-*β2t* gene including the 5’UTR was PCR amplified from genomic DNA of the wild type Italian strain using primer pair HM#35/HM#36 with program 98 °C 3′ [98 °C 30″, 61 °C 30″, 72 °C 30″] 5X [98 °C 30″,67 °C 30″72 °C 30″] 35X and cloned in *Nco*I/*Xba*I sites of mfs#1215 [10] giving rise to HMMA015. The *Dm-β2t* 3UTR was then PCR amplified from gDNA of wild type *D. melanogaster* strain OreR using primer pair HM#706/HM#707 with program 98 °C 3′ [98 °C 30″, 63 °C 30″, 72 °C 20″] 5X [98 °C 30″,70 °C 30″72 °C 20″] 35X and cloned into HMMA015 to give rise to HMMA253. Finally, the *Asc*I fragment from HMMA253 was cloned into the *Asc*I site of the transformation vector HMMA331.

To generate *attB* integration vector HMMA182 which can be used to integrate a plasmid into single *attP* site, the *5-piggyBac* region was PCR amplified from plasmid HMMA006 using primer pair T7/mfs#370, with program 98 °C 3′ [98 °C 30″, 51 °C 30″, 72 °C 20″] 40X digested by *Eco*RV and cloned into the blunted *BamH*I site of HMMA172, giving rise to HMMA181. Then the *Eco*RI/*Apa*I fragment containing the *PUb::nlsEGFP* was excised mfs#1213 [51] and cloned into *Eco*RI/*Apa*I of HMMA181.

To generate the helper plasmid HMMA098, the coding sequence of φC31 was PCR amplified from plasmid mfs#1289 [39] using primers pair MK153/HM#123 with program 98 °C 3′ [98 °C 30″, 72 °C 1′ 20″] 35X. The reverse primer introduces the *SV40 nuclear localization sequence* at the C-terminus, which can improve the efficiency of φC31 integrase [64]. A second round of PCR using primer pair MK153/HM#203 was used to amplify *φC31nls* using 1 μl of the first PCR reaction as a template with program 98 °C 3′ [98 °C 30″, 67 °C 30″, 72 °C 1′] 5X [98 °C 30″,72 °C 1′ 20″] 35X and clone into HMMA051 *Nco*I/*Not*I replacing the *piggyBac* transposase coding sequence and giving rise to HMMA098. The *piggyBac* helper HMMA051 was generated by cloning the *SV40* 3’UTR digested from CH#705 by *Hind*III/*Not*I into HMMA050 *Hind*III/*Not*I. sites. The latter was made by PCR amplification of *Ds-hsp70* promoter [52] from gDNA using primer pair HM73/HM#74 and program 98 °C 3’followed by [98 °C 30″, 58 °C 30″, 72 °C 30″] 5X [98 °C 30″, 66 °C 30″, 72 °C 30′] 35X and cloning into *Eco*RI site of HMMA049,which was generated by cloning the *piggyBac* transposase coding sequence excised from MK004 [23] by *Eco*RI/*Not*I into the shuttle vector pSLaf1180af [65].

To generate φC31 integrase based RMCE donor plasmids, HMMA253 and HMMA254, the annealed oligos HM#101/HM#337 generating the bacterial attachment site *attB* were cloned into *Spe*I site of pCRII vector (Invitrogene) giving rise to HMMA172. The *gypsy* insulators were digested out using *Spe*I/*Eco*RI from a fragment amplified from mfs#1213 [51] using primer pair HM#469/HM#470 with program 98 °C 3’followed by [98 °C 30″, 70 °C 30″, 72 °C 2′] 35X and cloned into the cut site of HMMA172 to give rise to HMMA189. The *Eco*RI/*Not*I fragments *PUb::nlsEGFP* and *PUb::DsRed.T3* were excised from HMMA186 and HMMA185, respectively, and cloned into HMMA189 to give rise to HMMA190 and HMMA191, respectively. Finally, *SV40* was PCR amplified from HMMA007 using primer pair HM#179/HM#124 and program 98 °C 3’followed by [98 °C 30″, 62 °C 30″, 72 °C 20″] 5X [98 °C 30″, 68 °C 30″, 72 °C 20″] 35X and cloned along with annealed oligos HM#101/HM#108 into HMMA190 and HMMA191 *Not*I/*Xba*I-blunted.

To generate HMMA336, for φC31-RMCE, the tetracycline responsive element TRE along with the *P-element* basal promoter was PCR amplified from CH 727 [9] using primers pair HM#584/ CH6R [9] with PCR program 98 °C 3’followed by [98 °C 30″, 69 °C 30″, 72 °C 30″] 35X and cloned into *Eco*RI/*Cla*I sites of HMMA56 [52] replacing the *hsp70* promoter giving rise to HMMA317 then the *Asc*I fragment containing *Cas9* fused to the *TREp* and the *SV40* 3’UTR was clone into *Asc*I site of HMMA253.

To generate self-docking transformation plasmid HMMA223 the *Asc*I fragment containing nosE/P-φC31-nos was excised from the shuttle vector HMMA221 and cloned into *Asc*I site of HMMA185. HMMA221 was generated by replacement of *Cas9* coding sequence in plasmid HMMA167 by *φC31 integrase* CDS. To make HMMA167, first the 3UTR of *Ds-nanos* was PCR amplified from HMMA013 using primer pair HM#94/HM95 with program 98 °C 3’followed by [98 °C 30″, 66 °C 30″, 72 °C 30] 5X [98 °C 30″, 72 °C 1′] 35X and cloned into the shuttle vector pSLaf1180af [65] *Xba*I/*Afl*II sites giving rise to HMMA062. Then *Cas9* CDS was excised from HMMA056 [52] and cloned into *Cla*I/*Xba*I sites of HMMA062 giving rise to HMMA165. Then the palindromic (self-complementary) oligo HM#102 was annealed to itself to introduce the 2X *Bbs*I recognition site and cloned into the *Cla*I site of HMMA165 to give rise to HMMA166. Finally, a 2 Kb upstream regulatory region of *Ds-nanos* gene including the 5’UTR was PCR amplified from gDNA using primer pair HM#345/HM#113 and program 98 °C 3’followed by [98 °C 30″, 72 °C 1′ 30″] 35X and cloned into HMMA166 *Bbs*I site by golden gate resulting in HMMA167.

### Germline transformation

All *piggyBac* germline transformation experiments were performed using transformation vector and helper plasmid MK006 [23] at a final concentration of 500 ng/μL and 200 ng/μL respectively. For φC31-mediated site-specific transformation and φC31-mediated RMCE, the donor vectors were injected along with the helper plasmid HMMA098 at a concentration of 500 ng/μL and 300 ng/μL, respectively. The materials and the procedure of germline transformation were as described previously [23, 52]. Emerged G_0_ flies were crossed individually to three wild type flies of the opposite sex.

### Generation of RNA probes

To generate DIG-labelled antisense RNA probes for in situ hybridization against *Ds_sryα, Ds_nanos, tTA*, *Cas9*, or *φC31 integrase*, DNA templates for in vitro transcription were prepared by restriction enzyme linearization of pCRII vectors containing either the whole gene pCRII_*Ds-sryα* (HMMA020), the 3’RACE fragment pCRII_*Ds-nos_3UTR* (HMMA013), the coding sequence pCRII_*tTA* (HMMA021), or 800 bp of the coding sequence of in case of pCRII_*Cas9* (FCMH01) and pCRII_φC31 (HMMA399) using *Xho*I*, Bam*HI*, Not*I, *Not*I, or *EcoR*I, respectively. The antisense RNA labelling reaction was done using the DIG-labelling kit (Thermo Fisher Scientific) according to manufacturer instructions using 1 μg of DNA as template in a total reaction mix of 20 μL. The reaction was allowed to proceed for 3 h at 37 °C followed by Turbo DNaseI treatment (Thermo Fisher Scientific) for 30 min to remove template DNA. Two microliter of 0.2 M EDTA were used to inactivate the reaction. The probes were then ethanol precipitated and resuspended in 100 μL RNA resuspension buffer (5:3:2 H_2_O: 20X SSC: formaldehyde) and stored at − 80 °C.

### Testes, ovary, and embryo whole mount in situ hybridization

Testes from 3 to 5 days old males from wild type *D. suzukii*, spermatogenesis specific driver line 389_M25M1, or progeny of the cross of the driver 389_M25M1 to the responder line 366_F3F1 were dissected in ice cold 1X phosphate buffered saline (PBS). Fixation and in situ hybridization were performed according to protocol by Lecuyer [66]. Anti-sense DIG labelled RNA probe against *tTA* was used to detect the expression driven by the *Ds-β2t E/P*. The *Cas9* anti-sense RNA probe was used to detect the expression of *Cas9* in the progenies arising from the cross testing the *tet-off* system. Anti-sense and sense probes previously described [52] were used as control.

To confirm the expression of the isolated *Ds-nanos* gene and the *φC31 integrase* driven by the regulatory regions of *Ds-nanos* in the ovaries of *D. suzukii* wild type flies and the transgenic self-docking line 223_F7M1, respectively, we collected 3–5 days old female flies and dissected the ovaries in ice-cold 1X PBS. The fixation and the in situ hybridization were performed as described [66].

To confirm the endogenous cellularization-specific expression of *Ds_sryα* in wild type embryos. and whether the 349 bp of its upstream regulatory region including the 5’UTR are enough to drive expression of *tTA* in the transgenic driver line 06_F5M2 in a similar pattern, we performed embryo whole mount in situ hybridization using respective anti-sense DIG-labelled RNA probes in 0–24 h old embryos. Fixation and in situ hybridization were performed according to Lecuyer [66].

### Microscopy

To observe and image testes, ovaries, and embryos, Zeiss Imager.Z2 equipped with two cameras, Axiocam 506 mono and Axiocam 305 colour (Zeiss, 73447 Oberkochen, Germany) was used. Images were taken using Axiocam 305 with bright field or DIC settings.

Screening for transgenic flies and fluorescence imaging were performed using Leica M205 FA fluorescence stereomicroscope equipped with camera Q imaging Micropublisher 5.0 RTV (Leica Mikrosysteme Vertrieb Gmb, Wetzlar, 35578 Germany). Transgenic flies were screened using filter sets RFP (excitation: ET546/10, emission: ET605/70) or GFP-LP (excitation: ET480/40, emission: ET510 LP), respectively, and imaged using cold light (Fig. 3A′,B′), filter sets RFP (Fig. 3A″,B″), or EYFP (excitation: ET500/20, emission: ET535/30; Fig. 3A‴,B‴).

## Supplementary Information

**Additional file 1:** **Supplementary Table 1.** *piggyBac* transformation rates in *D. suzukii* AM strain.

**Additional file 2:** **Supplementary Table 2.** List of transgenic lines.

**Additional file 3:** **Supplementary Table 3.** List of primers used.

## Data Availability

All data generated or analysed during this study are included in this published article and its supplementary information files.

## Abbreviations

φC31-RMCE: φC31-Recombinase Mediated Cassette Exchange
*Ds nanos*: *Drosophila suzukii nanos*
*tTA*: *Tetracycline-controlled transactivator*
*β2t*: *Beta-2-tubulin*
SWD: The spotted wing *Drosophila*
SIT: Sterile Insect Technique
SSR: Site-specific recombinases
BAC: Bacterial artificial chromosome
dsRNA: Double strand RNA
*hid*: *Head involution defective*
AM: Alpes Maritimes
*sry α*: *Serendipity α*
*Dm_sry α*: *Drosophila melanogaster serendipity α*
*Ds_sry α*: Drosophila suzukii *serendipity α*
DsRed: Discosoma Red
EGFP: Enhanced green fluorescent protein
*Cas9*: CRISPR associated protein 9
*Dm_nanos*: *Drosophila melanogaster nanos*
DIG: Digoxigenin
3′ UTR: 3′ untranslated region
5’UTR: 5’untranslated region
cDNA: Complementary DNA
RACE: Rapid amplification of cDNA ends
PBS: Phosphate buffered saline
DIC: Differential interference contrast

## References

[1] Hauser M (2011). A historic account of the invasion of Drosophila suzukii (Matsumura) (Diptera: Drosophilidae) in the continental United States, with remarks on their identification. Pest Manag Sci.

[2] Walsh DB, Bolda MP, Goodhue RE, Dreves AJ, Lee J, Bruck DJ (2011). Drosophila suzukii (Diptera: Drosophilidae): invasive Pest of ripening soft fruit expanding its geographic range and damage potential. J Integrated Pest Manag.

[3] Cini A, Ioriatti C, Anfora G (2012). A review of the invasion of Drosophila suzukii in Europe and a draft research agenda for integrated pest management. B Insectol J.

[4] Mazzi D, Bravin E, Meraner M, Finger R, Kuske S (2017). Economic impact of the introduction and establishment of Drosophila suzukii on sweet cherry production in Switzerland. Insects..

[5] Haviland DR, Beers EH (2012). Chemical Control Programs for Drosophila suzukii that Comply With International Limitations on Pesticide Residues for Exported Sweet Cherries. J Integ Pest Manage.

[6] Van Timmeren S, Isaacs R (2013). Control of spotted wing drosophila, Drosophila suzukii, by specific insecticides and by conventional and organic crop protection programs. Crop Prot.

[7] Knipling EF (1955). Possibilities of insect control or eradication through the use of sexually sterile males. J Econ Entomol.

[8] Heinrich JC, Scott MJ (2000). A repressible female-specific lethal genetic system for making transgenic insect strains suitable for a sterile-release program. Proc Natl Acad Sci.

[9] Horn C, Wimmer EA (2003). A transgene-based, embryo-specific lethality system for insect pest management. Nat Biotechnol.

[10] Schetelig MF, Caceres C, Zacharopoulou A, Franz G, Wimmer EA (2009). Conditional embryonic lethality to improve the sterile insect technique in Ceratitis capitata (Diptera: Tephritidae). BMC Biol.

[11] Ogaugwu CE, Schetelig MF, Wimmer EA (2013). Transgenic sexing system for Ceratitis capitata (Diptera: Tephritidae) based on female-specific embryonic lethality. Insect Biochem Mol Biol.

[12] Yan Y, Scott MJ (2015). A transgenic embryonic sexing system for the Australian sheep blow fly Lucilia cuprina. Sci Rep.

[13] Concha C, Palavesam A, Guerrero FD, Sagel A, Li F, Osborne JA (2016). A transgenic male-only strain of the New World screwworm for an improved control program using the sterile insect technique. BMC Biol.

[14] Schetelig MF, Handler AM (2012). A transgenic embryonic sexing system for Anastrepha suspensa (Diptera: Tephritidae). Insect Biochem Mol Biol.

[15] Woltjen K, Michael IP, Mohseni P, Desai R, Mileikovsky M, Hämäläinen R (2009). piggyBac transposition reprograms fibroblasts to induced pluripotent stem cells. Nature.

[16] Wang W, Lin C, Lu D, Ning Z, Cox T, Melvin D (2008). Chromosomal transposition of PiggyBac in mouse embryonic stem cells. Proc Natl Acad Sci.

[17] Park MA, Jung HS, Slukvin I (2018). Genetic engineering of human pluripotent stem cells using PiggyBac transposon system. Curr Protoc Stem Cell Biol.

[18] Cary LC, Goebel M, Corsaro BG, Wang HG, Rosen E, Fraser MJ (1989). Transposon mutagenesis of baculoviruses: analysis of Trichoplusia ni transposon IFP2 insertions within the FP-locus of nuclear polyhedrosis viruses. Virology..

[19] Hacker U, Nystedt S, Barmchi MP, Horn C, Wimmer EA (2003). piggyBac-based insertional mutagenesis in the presence of stably integrated P elements in Drosophila. Proc Natl Acad Sci.

[20] Handler AM (2002). Use of the piggyBac transposon for germ-line transformation of insects. Insect Biochem Mol Biol.

[21] Handler AM, Harell RA. Germline transformation of *Drosophila melanogaster* with the piggyBac transposon vector. Insect Mol Biol. 1999;8:499–57. 10.1046/j.1365-2583.1999.00139.x 10634970

[22] Handler AM, McCombs SD, Fraser MJ, Saul SH (1998). The lepidopteran transposon vector, piggyBac, mediates germ-line transformation in the Mediterranean fruit fly. Proc Natl Acad Sci.

[23] Eckermann KN, Ahmed HMM, KaramiNejadRanjbar M, Dippel S, Ogaugwu CE, Kitzmann P (2018). Hyperactive piggyBac transposase improves transformation efficiency in diverse insect species. Insect Biochem Mol Biol.

[24] Condon KC, Condon GC, Dafa’alla TH, Forrester OT, Phillips CE, Scaife S (2007). Germ-line transformation of the Mexican fruit fly. Insect Mol Biol.

[25] Schetelig MF, Handler AM (2013). Germline transformation of the spotted wing drosophilid, Drosophila suzukii, with a piggyBac transposon vector. Genetica..

[26] Grossman GL, Rafferty CS, Clayton JR, Stevens TK, Mukabayire O, Benedict MQ (2001). Germline transformation of the malaria vector, Anopheles gambiae, with the piggyBac transposable element. Insect Mol Biol.

[27] Lobo NF, Hua-Van A, Li X, Nolen BM, Fraser MJ (2002). Germ line transformation of the yellow fever mosquito, Aedes aegypti, mediated by transpositional insertion of a piggyBac vector. Insect Mol Biol.

[28] Hediger M, Niessen M, Wimmer EA, Dübendorfer A, Bopp D (2001). Genetic transformation of the housefly Musca domestica with the lepidopteran derived transposon piggyBac. Insect Mol Biol.

[29] Bonin CP, Mann RS (2004). A piggyBac transposon gene trap for the analysis of gene expression and function in Drosophila. Genetics..

[30] O’Brochta DA, Alford RT, Pilitt KL, Aluvihare CU, Harrell RA (2011). piggyBac transposon remobilization and enhancer detection in Anopheles mosquitoes. Proc Natl Acad Sci.

[31] Gayle S, Pan Y, Landrette S, Xu T (2015). piggyBac Insertional mutagenesis screen identifies a role for nuclear RHOA in human ES cell differentiation. Stem Cell Rep.

[32] Henikoff S (1992). Position effect and related phenomena. Curr Opin Genet Dev.

[33] Levis R, Hazelrigg T, Rubin GM (1985). Effects of genomic position on the expression of transduced copies of the white gene of Drosophila. Science..

[34] Siegal ML, Hartl DL (1996). Transgene Coplacement and high efficiency site-specific recombination with the Cre/loxP system in Drosophila. Genetics..

[35] Long D-P, Zhao A-C, Chen X-J, Zhang Y, Lu W-J, Guo Q (2012). FLP Recombinase-mediated site-specific recombination in silkworm, *Bombyx mori*. PLoS ONE.

[36] Turan S, Galla M, Ernst E, Qiao J, Voelkel C, Schiedlmeier B (2011). Recombinase-mediated cassette exchange (RMCE): traditional concepts and current challenges. J Mol Biol.

[37] Turan S, Bode J. Site-specific recombinases: from tag-and-target- to tag-and-exchange-based genomic modifications. FASEB J. 2011. 10.1096/fj.11-186940. 10.1096/fj.11-186940 21891781

[38] Bode J, Schlake T, Iber M, Schübeler D, Seibler J, Snezhkov E (2005). The Transgeneticists toolbox: novel methods for the targeted modification of eukaryotic genomes. Biol Chem.

[39] Schetelig MF, Scolari F, Handler AM, Kittelmann S, Gasperi G, Wimmer EA (2009). Site-specific recombination for the modification of transgenic strains of the Mediterranean fruit fly Ceratitis capitata. Proc Natl Acad Sci.

[40] Wimmer EA (2005). Insect transgenesis by site-specific recombination. Nat Methods.

[41] Venken KJT, He Y, Hoskins RA, Bellen HJ (2006). P[acman]: A BAC Transgenic Platform for Targeted Insertion of Large DNA Fragments in *D. melanogaster*. Science.

[42] Bischof J, Maeda RK, Hediger M, Karch F, Basler K (2007). An optimized transgenesis system for Drosophila using germ-line-specific φC31 integrases. PNAS..

[43] Meredith JM, Underhill A, McArthur CC, Eggleston P (2013). Next-generation site-directed Transgenesis in the malaria vector mosquito Anopheles gambiae: self-docking strains expressing Germline-specific phiC31 Integrase. PLoS One.

[44] Leulier F, Vidal S, Saigo K, Ueda R, Lemaitre B (2002). Inducible expression of double-stranded RNA reveals a role for dFADD in the regulation of the antibacterial response in Drosophila adults. Curr Biol.

[45] Matsushima Y, Adán C, Garesse R, Kaguni LS, Leister D, Herrmann JM (2007). Functional analysis by inducible RNA interference in Drosophila melanogaster. Mitochondria.

[46] Gossen M, Bujard H (1992). Tight control of gene expression in mammalian cells by tetracycline-responsive promoters. Proc Natl Acad Sci.

[47] Urlinger S, Baron U, Thellmann M, Hasan MT, Bujard H, Hillen W (2000). Exploring the sequence space for tetracycline-dependent transcriptional activators: novel mutations yield expanded range and sensitivity. Proc Natl Acad Sci.

[48] Zhou X, Vink M, Klaver B, Berkhout B, Das AT (2006). Optimization of the Tet-On system for regulated gene expression through viral evolution. Gene Ther.

[49] Fu G, Condon KC, Epton MJ, Gong P, Jin L, Condon GC (2007). Female-specific insect lethality engineered using alternative splicing. Nat Biotechnol.

[50] Catteruccia F, Benton JP, Crisanti A (2005). An Anopheles transgenic sexing strain for vector control. Nat Biotechnol.

[51] Scolari F, Schetelig MF, Bertin S, Malacrida AR, Gasperi G, Wimmer EA (2008). Fluorescent sperm marking to improve the fight against the pest insect Ceratitis capitata (Wiedemann; Diptera: Tephritidae). New Biotechnol.

[52] Ahmed HMM, Hildebrand L, Wimmer EA (2019). Improvement and use of CRISPR/Cas9 to engineer a sperm-marking strain for the invasive fruit pest Drosophila suzukii. BMC Biotechnol.

[53] Eckermann KN, Dippel S, KaramiNejadRanjbar M, Ahmed HM, Curril IM, Wimmer EA (2014). Perspective on the combined use of an independent transgenic sexing and a multifactorial reproductive sterility system to avoid resistance development against transgenic Sterile Insect Technique approaches. BMC Genet.

[54] Karageorgi M, Bräcker LB, Lebreton S, Minervino C, Cavey M, Siju KP (2017). Evolution of multiple sensory systems drives novel egg-laying behavior in the fruit pest Drosophila suzukii. Curr Biol.

[55] Handler AM, Zimowska GJ, Horn C (2004). Post-integration stabilization of a transposon vector by terminal sequence deletion in Drosophila melanogaster. Nat Biotechnol.

[56] Liebl FLW, Werner KM, Sheng Q, Karr JE, McCabe BD, Featherstone DE (2006). Genome-wideP-element screen forDrosophila synaptogenesis mutants. J Neurobiol.

[57] Bachmann A, Knust E, Dahmann C (2008). The use of P-element transposons to generate transgenic flies. Drosophila.

[58] Venken KJT, Bellen HJ (2005). Emerging technologies for gene manipulation in Drosophila melanogaster. Nat Rev Genet.

[59] Rio DC, Rubin GM. Identification and purification of a Drosophila protein that binds to the terminal 31-base-pair inverted repeats of the P transposable element. Proc Natl Acad Sci USA. 1988;85:8929–33. 10.1073/pnas.85.23.8929 PMC282620 2848246

[60] Brown SJ, Shippy T, Miller S, Bolognesi R, Beeman RW, Lorenzen MD (2009). The red flour beetle, *Tribolium castaneum* (Coleoptera): a model for studies of development and pest biology. Cold Spring Harbor Protoc.

[61] Berghammer AJ, Klingler M, Wimmer EA (1999). A universal marker for transgenic insects. Nature..

[62] Buchman A, Marshall JM, Ostrovski D, Yang T, Akbari OS (2018). Synthetically engineered *Medea* gene drive system in the worldwide crop pest *Drosophila suzukii*. Proc Natl Acad Sci U S A.

[63] Schetelig MF, Yan Y, Zhao Y, Handler AM (2019). Genomic targeting by recombinase-mediated cassette exchange in the spotted wing drosophila, Drosophila suzukii. Insect Mol Biol.

[64] Andreas S, Schwenk F, Küter-Luks B, Faust N, Kühn R (2002). Enhanced efficiency through nuclear localization signal fusion on phage PhiC31-integrase: activity comparison with Cre and FLPe recombinase in mammalian cells. Nucleic Acids Res.

[65] Horn C, Wimmer EA (2000). A versatile vector set for animal transgenesis. Dev Gene Evol.

[66] Lécuyer E, Gerst JE (2011). High resolution fluorescent in situ hybridization in Drosophila. RNA detection and visualization.

