# Supplementary material for: Improvement on the genetic engineering of an invasive agricultural pest insect, the cherry vinegar fly, Drosophila suzukii
Source: BMC Genet. 2020 Dec 18;21(Suppl 2):139. doi: 10.1186/s12863-020-00940-5 (PMC7747376; doi:10.1186/s12863-020-00940-5)
Supplement: Supplementary file 1 — Additional file 1: Supplementary Table 1. piggyBac transformation rates in D. suzukii AM strain. [file 12863_2020_940_MOESM1_ESM.pdf]

Additional File 1

Supplementary Table 1: *piggyBac* transformation rates in *D. suzukii* AM strain

Construct	No injected embryos	Hatched larvae	Fertile crosses	No. transgenics	Transformation rate in %
HMMA389	350	185	40	1	2.5
HMMA185	475	181	45	2*	4.4
HMMA223	290	150	18	2	11

* For one transgenic F_1, no line could be established.
