# Supplementary material for: Improvement on the genetic engineering of an invasive agricultural pest insect, the cherry vinegar fly, Drosophila suzukii
Source: BMC Genet. 2020 Dec 18;21(Suppl 2):139. doi: 10.1186/s12863-020-00940-5 (PMC7747376; doi:10.1186/s12863-020-00940-5)
Supplement: Supplementary file 2 — Additional file 2: Supplementary Table 2. List of transgenic lines. [file 12863_2020_940_MOESM2_ESM.pdf]

Additional File 2.

Supplementary Table 2: List of transgenic lines

Strategy	Construct	Transgenic lines
piggyBac	HMMA006 (embryonic driver, *attP*), Figs. 1B; 3A	06_F5M2 (attP#1)
	HMMA389 (spermatogenesis driver), Fig. 2A	389_F25M1
	HMMA185 (φC31 RMCE docking), Fig. 3B	185_F3F1 (RMCE#1)
	HMMA223 (φC31 RMCE self-docking), Fig. 4C	223_M3M2 (RMCE-sd#1)<br>223_F7M1 (RMCE-sd#2)<br>223_F5F1 (RMCE-sd#3)<br>223_M10F1 (RMCE-sd#4)
***φC31*-int**	HMMA182 (single *attB* donor), Fig. 3A<br>Injected into 06_F5M2 (attP#1)	attP#1_182_M12F1<br>attP#1_182_F8M1<br>attP#1_182_F15F1<br>attP#1_182_F25M1
***φC31*-RMCE**	HMMA336 (RMCE donor TREp:Cas9), Fig. 3B<br>Injected into 185_F3F1 (RMCE#1)	RMCE#1_336_M1M1<br>RMCE#1_336_M17F1<br>RMCE#1_336_M21M1<br>RMCE#1_336_M32M1<br>RMCE#1_336_M33F1<br>RMCE#1_336_M34M3<br>RMCE#1_336_F12F1<br>RMCE#1_336_F3F2
