# Supplementary material for: Improvement on the genetic engineering of an invasive agricultural pest insect, the cherry vinegar fly, Drosophila suzukii
Source: BMC Genet. 2020 Dec 18;21(Suppl 2):139. doi: 10.1186/s12863-020-00940-5 (PMC7747376; doi:10.1186/s12863-020-00940-5)
Supplement: Supplementary file 3 — Additional file 3: Supplementary Table 3. List of primers used. [file 12863_2020_940_MOESM3_ESM.pdf]

Additional File 3

Supplementary Table 3: List of primers used

Code	Name	Sequence 5'- 3'
**HM#16**	sryaR4	TTGTGTGTCATGGATGTTCAATCTAATC
**HM#17**	Srya5UTR_F1	GTACTTAGTTGAAAAGTTCAGCTTTACCCG
**HM#34**	Ds_srya_GSP3	GGCATCCAGGCTAATGGTCCGCTCCAAGTG
**HM#35**	B2t_NcoI_F1	GCAACCATGGGATGCCAAGAGAGATGAGCAGG
**HM#36**	B2t_XbaI_R3	CGATTCTAGACATCTTAACCGACTGTCAAGGATC
**HM#42**	srya_GSP_F	GCCTCTCTGGCTCCGATTCCCCCTAATG
**HM#73**	hspEcoRIF2	GCGAATTCTCCAGAAACTCAAACAGAAACC
**HM#74**	hspEcoRIR	GCGAATTCTTGTGTGTTTGTGTTTGTGGATGCAG
**HM#76**	nos_GSPR	GAGTCCTCCTCTTGCGTGGAATGCCG
**HM#77**	nos_GSPF	GTACTGTCCCAAGAAGCCGATTATCAC
**HM#94**	nos3UTRXbaIF	GGTCTAGAGAACACATCCGGCAGGAGC
**HM#95**	nos3UTRAflIIR	ATACTTAAGACTGAGCTCCAAGCAGTGGTATCAACGCAGAG
**HM#101**	SpeI-atBF	CTAGTGTGAGGTGGAGTACGCGCCCGGGGAGCCCAAGGGCACGCCCTGGCACCCGCAC
**HM#102**	BbsI-BbsI	CGGTCTTCGCGAAGAC
**HM#113**	BbsI_nosR	GCGAAGACCCATATGGCGAAAGTCCGGCTCGAAAGTTACC
**HM#117**	HM_Pub_R	CATTGGAATCTCTGTCGCTGCGTTCCG
**HM#123**	phi_nls_R	CTAGACCTTCCGCTTCTTCTTTGGGGCCGCCGCTACGTCTTCCGTGCCGTCCTG
**HM#124**	SV40_SpeI_R	CACACTAGTGATACATTGATGAGTTTGGACAAACCACAAC
**HM#131**	PLF2	GTCAAAATGACGCATGATTATCTTTTACG
**HM#179**	DsRed_End	CGAGGGCCGCCACCACCTGTTCCTG
**HM#203**	NotI_phiC31_R	TCGCGGCCGCCTAGACCTTCCGCTTCTTCTTTGG
**HM#337**	SpeI_attB_R	CTAGGTGCGGGTGCCAGGGCGTGCCCTTGGGCTCCCCGGGCGCGTACTCCACCTCACA
**HM#345**	nosP_Bbsi_F	CGGAAGACCGCGATTCCTTCAGTATCTCCAAATCGCCCCGGAC
**HM#368**	attP220_Fwd	TCATCAATGTATCACTAGTACTGACGGACACACC
**HM#369**	attP220_Rev	CTGGCTGGGGAATCTGTACTAGTCGCGCTCG
**HM#469**	SpeI_gypsy_F	GCTTACTAGTGATGGTCTCAAGCTTGTCAGATCGGC
**HM#470**	ApaI_SV40_R	TTAGGGCCCCGCCTTAAGATACATTGATGAGTTTGG
**HM#560**	FH_Cas9_HidIII_F	CATCAAGCTTACAAGTTCATCAAGCCCATCCTGG
**HM#561**	FH_Cas9_XhoI_R	CATGCTCGAGATAGGTTTTCAGCCGTTCCTCGATC
**HM#584**	HM_EcoRI_TRE_F2	TACGAATTCGGCGCGCCTAGGCCGGCCGAATTTC
**HM#706**	HM_b2t_3UTR_F	CGAGGATCCTAGGATTAACTTCCCACTCAAGATCACACATG
**HM#707**	HM_b2t_3UTR_R	GCCAAGCTTGTCTGCTTATAAATCAACATTTATTCGTAACCC
**mfs#370**	AflII-5pBac_F	AACTTAAGTTAACCCTAGAAAGATAGTCTGC
**MK153**	PhiC31_BsaI_F	ATGGTCTCACATGGACACGTACGCGGGTGCTTACGAC
**T7**	T7	TAATACGACTCACTATAGGG
**CH6R**	CH_3'PIClaI_2	CCATCGATGGAATGAACAGGACCTAACGC
